# *Pseudomonas* ST1 and *Pantoea* Paga Strains Cohabit in Olive Knots

**DOI:** 10.3390/microorganisms10081529

**Published:** 2022-07-28

**Authors:** Gabriela Vuletin Selak, Marina Raboteg Božiković, Danis Abrouk, Marija Bolčić, Katja Žanić, Slavko Perica, Philippe Normand, Petar Pujic

**Affiliations:** 1 Institute for Adriatic Crops and Karst Reclamation, Put Duilova 11, 21000 Split, Croatia; marina.raboteg@krs.hr (M.R.B.); marija.mandusic@krs.hr (M.B.); katja.zanic@krs.hr (K.Ž.); slavko.perica@krs.hr (S.P.); 2 Centre of Excellence for Biodiversity and Molecular Plant Breeding (CoE CroP-BioDiv), Svetošimunska Cesta 25, 10000 Zagreb, Croatia; 3 Ecologie Microbienne, Centre National de la Recherche Scientifique UMR 5557, Université de Lyon, Université Claude Bernard Lyon I, INRAE, UMRA1418, 69622 Villeurbanne, France; danis.abrouk@univ-lyon1.fr (D.A.); philippe.normand@univ-lyon1.fr (P.N.)

**Keywords:** host pathogen interaction, *Pseudomonas* ST1, *Pantoea* paga, galls, *Olea europaea* L., plant microbiome, antimicrobial peptides

## Abstract

Two bacteria belonging to the *Pseudomonas* and *Pantoea* genera were isolated from olive knots. Both bacterial strains were omnipresent in this study’s olive orchard with high susceptibility of the autochthonous olive genotypes indicating coevolution of bacteria with host plants. Genomes of two endemic bacteria show conserved core genomes and genome plasticity. The *Pseudomonas* ST1 genome has conserved virulence-related genes including genes for quorum sensing, pilus, and flagella biosynthesis, two copies of indole acetic acid biosynthesis (IAA) operons, type I-VI secretions systems, and genes for alginate and levan biosynthesis. Development of knots depends only on the presence of the *Pseudomonas* ST1 strain which then allows *Pantoea* paga strain co-infection and cohabitation in developed knots. The two bacteria are sensitive to a large number of antimicrobials, antibiotics, H_2_O_2_, and Cu (II) salts that can be efficiently used in propagation of bacterial free olive cultivars.

## 1. Introduction

Olive knot disease caused by the Gram-negative bacterium *Pseudomonas savastanoi* pv. *savastanoi* is recognized as one of the most important diseases that affect olive trees (*Olea europaea* L.) throughout various regions of the world, in particular the Mediterranean region [1]. *Pseudomonas savastanoi* belongs to the *Pseudomonas syringae* complex and includes three pathogenic lineages that cause tumorous overgrowths (tumorous galls or knots) in diverse trees and shrubs of economic relevance. In addition to *Pseudomonas savastanoi* pv. *savastanoi*, other pathovars such as *P. savastanoi* pv. *nerii* and *P. savastanoi* pv. *fraxini* have been isolated from oleander and ash, respectively.

*Pseudomonas savastanoi* pv. *savastanoi* (Psv, according to [2]) lives mainly as an epiphyte on the surface of olive organs [3]. Under favorable weather conditions, its population increases and the bacteria may enter into olive tissues and survive as an endophyte. Psv may experience uncontrolled proliferation at the site of infection and form knots trig-gered by phytohormones synthesized by the bacterium [1,4,5]. The knots occur mainly on the stems and branches of the host plants, and more rarely on leaves and fruits [6]. However, there is limited information on the impacts of this disease on tree vigor and yield [7].

At short distances, the bacterium can spread by rain, windblown aerosols, insects and agricultural practices. Processes such as the falling of leaves, budding, harvesting and pruning create wounds on the plant through which infection occurs [8]. Weather conditions favorable for both bacterium epiphytic development and its entry into the host determine the extent of damages caused by Psv. Olive knots are optimal niches for Psv growth and also for other bacteria. For example, many bacterial species reside inside the knots together with *Pseudomonas savastanoi* pv. *savastanoi*, commonly of the genera *Pantoea*, *Pectobacterium*, *Erwinia* and *Curtobacterium* [9]. Previous studies have reported that the microbe interactions can result in enhanced or decreased pathogenicity of one bacterium towards their plant host. Likewise, the interaction of Psv with other bacterial species has shown to mediate the disease potential [10,11]. Several non-pathogenic bacterial species, namely *Erwinia toletana*, *Pantoea agglomerans,* and *Erwinia oleae*, can form a stable bacterial consortium with the pathogen Psv and increase disease severity by boosting the proliferation of olive tissues [9,12,13]. The structure of plant-associated microbiota is genotype dependent [14,15] and may lead to different pathobiomes [16].

The pathogenicity and virulence of bacteria from the *Pseudomonas syringae* group and olive isolates of *P. savastanoi* are dependent on a type III secretion system (T3SS) that has been identified as a key virulence determinant among many Gram-negative plant and animal pathogenic bacteria [17,18,19,20]. Bacteria of the *P. syringae* group cause disease by suppressing plant defense responses after delivering effector proteins into the host cell cytoplasm using a type III secretion system (T3SS) [21]. Sisto et al. [17] reported that T3SS is encoded by *hrp*/*hrc* gene clusters. The T3SS is required for the hypersensitive response and for pathogenicity [22]. In addition to effector proteins, phytotoxins and hormones are recognized as molecules that increase virulence or microbial fitness [18,23,24,25,26]. The phytohormones indoleacetic acid (IAA) and cytokinins (CK) are the first and best-characterized Psv determinants that have been shown to be involved in olive knot formation [5,27].

Due to the economic impact of olive knot disease, growers need effective control methods to overcome its negative effects on plant growth and fruit yield [28]. Traditional control strategies are mainly based on reducing the endophytic and epiphytic *P. savastanoi* populations with practices that include pruning of affected branches and treating with copper compounds [28]. The use of resistant olive cultivars is considered one of the most efficient methods of disease control but limited information is available from these comparative studies [29,30,31]. Although different olive cultivars have shown different susceptibility to olive knot disease, there exists no report of olive tree genotypes completely resistant to disease [11]. To avoid the spread of *P. savastanoi* or other pathogens, sanitary certification programs for olive propagation materials and mother plants have been already launched worldwide [32]. However, the measures for the production of certified olive plants are sometimes based on the visual inspection of the knots, without considering the possible asymptomatic presence of *P. savastanoi*.

The plant genotype has been increasingly recognized to be a key determinant of phyllosphere microbiota composition [14]. The aim of this work was to evaluate the susceptibility of olive germplasm to olive knot disease caused by *Pseudomonas* and *Pantoea*, including isolation of the causative agent of knot formation and confirmation of pathogenicity by inoculation of healthy plants with isolated bacterial strains.

The genus *Pantoea* is a member of the Enterobacteriaceae and includes several species [33]. Most of them are associated with many plants living as a part of plants epiphyte or endophyte microbiome. Some strains are considered as plant growth promoting bacteria (PGPB). *Pantoea aglomerans* strains evolved into pathogens that cause gall formation on various plants [34,35] and another prevents root development in pruned cuttings as a limiting factor in plant propagation [36]. In this work we describe the effects of two bacteria in forming galls on olive plants and determine the genomic potential, phylogeny, and virulence of each strain.

## 2. Materials and Methods

### 2.1. The Incidence of Olive Knot Disease in Different Olive Cultivars

The study was carried out in 2019 in the collection orchard of olive cultivars from the main olive producing countries of the world and of the most common nationally planted cultivars of Croatia. The collection orchard is situated in Kaštela (43°3′22″ N, 16°20′56″ E). It consists of four Croatian cultivars, ‘Oblica‘, ‘Lastovka’, ‘Levantinka’ and ‘Drobnica’. In addition to the aforementioned national cultivars, the collection preserves cultivars from Italy (Canino, Coratina, Fasolina, Favarol, Frantoio, Grignan, Leccino, Maurino, Moraiolo, Pendolino, Rosciola, Santa Catarina, Taggiasca), Greece (Koroneiki), Algeria (Sigoise), Tunisia (Chemlali) and Morocco (Picholine marocaine).

In November 2019, the symptoms caused by olive knot in different olive cultivars were evaluated, five months after a hailstorm caused great damage to the trees of all cultivars in the collection. The evaluation of the symptoms was carried out according to the proposed methodology for the secondary characterization of olive varieties held in collections. The incidence of the disease was assessed by visual inspection of at least four trees per cultivar. The percentage of area in which symptoms appear was evaluated and cultivars were classified into six different categories of tolerance corresponding to percentages (1. Nil: 0; 2. Very low: 1–20%; 3. Low: 20–40%; 4. Medium: 40–60%; 5. High: 60–80%; 6. Very high: 80–100%).

### 2.2. Genetic and Phylogenetic Analysis of Pseudomonas ST1 and Pantoea Paga 

Two genomes, *Pseudomonas* ST1 and *Pantoea* paga strains have been sequenced earlier and deposited under the accession numbers VKOF00000000.1 [37] and VLTF00000000 [38], respectively. Genomes of *Pseudomonas* ST1 and *Pantoea* paga strains were analyzed on the Mage platform [39] to compute clusters of orthologous genes (COGs) [40]. Using antiSMASH [41] genes clusters involved in the biosynthesis of secondary metabolites and virulence factors [42] were identified.

Using GBDP distances calculated from 16S rDNA gene sequences, phylogenetic trees were created with FastME 2.1.6.1. The branch lengths are scaled in terms of GBDP distance formula d5. The numbers above branches are GBDP pseudo-bootstrap support values > 60% from 100 replications, with an average branch support of 52.6%. The tree was rooted at the midpoint [43,44].

### 2.3. Confirmation of Pathogenicity of Pseudomonas ST1 and Pantoea Paga Isolated from the Trees in a Collection Orchard

The experiment was performed in an unshaded greenhouse at the Institute for Adriatic Crops and Karst Reclamation, Split in October 2020. Two olive cultivars Oblica and Lastovka that showed significantly different susceptibilities to olive knot disease in the first experiment were included in the study. Plants were planted in 5 L pots with a mixture of substrates including “Brill TYPical 4” and eutric brown soil (pH 5.5–6.8; 2–6% humus content). With a sterile scalpel, lateral wounds, approximately 5 to 10 mm in diameter, were introduced to the shoots of the two-year-old plants. The cambial tissue was exposed to artificial infection performed by inoculation with *Pseudomonas* ST1 strain [37], *Pantoea* paga strain [38], and combination of these two bacteria. Replicates of five plants per cultivar with ten lateral wounds was used (in total, fifty wounds per treatment condition and cultivar). In control plants no inoculation with bacterial strains was performed. Olive trees were assessed 6 months after inoculation and the percentage of knot formation was calculated for each treatment (number of wounds with symptoms/total number of wounds × 100). Treatments were evaluated based on the incidence of disease. The data for knot formation in different treatments were subjected to an analysis of variance using generalised linear models in SAS software (SAS Institute Inc., Cary, NC, USA). Differences between groups were determined by the LSD test at *p* ≤ 0.05.

To confirm the presence of bacteria in lateral wounds, bacteria from the olive knots and plant tissue around the wounds were cultured from the plants that showed symptoms of infection as well as from the plants that did not develop symptoms including control plants. The samples were sterilized using 75% ethanol, sliced with a sterile scalpel, and put onto King’s medium agar plates, kept in the dark [45]. The bacterial cells developed on medium were transferred onto a new King’s medium agar plates and kept at 37 °C. An isolated single colony from each treatment was transferred into 5 ml of liquid lysogeny broth (LB) medium and grown at 28 °C. DNA was isolated from bacterial cells in the precipitate using a microbial DNA kit (Macherey-Nagel, Hoerdt, France). 16S RNA gene was amplified in each sample using PCR reaction and sequenced.

#### The Efficiency of the Primers in Detection of the Bacteria

‘Lastovka’, the cultivar with the highest intensity symptoms, was used for virulence determination. Olive knots and plant tissue around the inoculation sites for the two bacterial strains were taken from plant shoots and disassociated with a sterile scalpel. The control plants were also treated similarly. The DNA isolation from disease knots and control plant tissue was purified with a microbial DNA kit (Macherey-Nagel, Hoerdt, France). The concentration of the DNA was measured spectrophotometrically by UV absorbance at 260–280 nm. The PCR amplifications were performed using sets of specific primers. *Pantoea* primers were: paga20853f 5′-TTACCCAGCGTGAAACCCG, paga20853r 5′-TCGGCTGGGTTCAGGCTC, paga20492f 5′-GGCCAAAGAAGACAATATTGAA, paga20492r 5′-AGCGACTACGGAAGACAATG, paga20832f 5′-TGGTACTGATTGCAGCGGG, paga20832r 5′-GCCCTTTGCCATCCTCCG. *Pseudomonas* ST1 primers were: PSST1-1220002f 5′-TCGGGGACCTGCACGTCC, PSST1-1220002r 5′-AGCGCCCTGGCCTACTGG, PSST1-1220003f 5′-ACTACCAGCTCCCAGGGC, PSST1-1220003r 5′-GGCGCCATCAGAGCCATAC. For 16sRNA primers were: 16S27f 5′-AGAGTTTGATCMTGGCTCAG, 16S1492r 5′-GGYTWCCTTGTTACGACTT [46,47], pA 5′-AGAGTTTGATCCTGGCTCAG, pH 5′-AAGGAGGTGATCCAGCCGCA [48], com1 CAGCAGCCGCGGTAATAC and com2 CCGTCAATTCCTTTGAGTTT [49]. DNA amplification was done in 25 µL volume of reaction mixture containing specific primers, 1× reaction buffer, dNTPs, DNA template and Taq polymerase (NEB) in accordance with manufacturer’s instructions with the following conditions: 95 °C for 5 min; 35 cycles of 95 °C for 20 s, 55 °C for 45 s and 72 °C for 1 min.

### 2.4. Susceptibility of Bacteria to Antibiotic and Chemical Agents Tested by a Standardized Single Disk Method

#### 2.4.1. Bacteria Strains

The strains of two bacterial species used in this study were from our culture collection. *Pseudomonas* ST1 and *Pantoea* paga cells were isolated from olive knots from olive plants grown in the central region of Dalmatia, Croatia. The complete genome sequence was described and has been deposited in NCBI/GenBank [37,38].

#### 2.4.2. Growth Media, Antibiotics and Chemical Agents

King’s A media for a single-disc diffusion technique was prepared according to King et al. [45]. The medium was used for antibiotic and chemical agents’ susceptibility testing. A single-disc diffusion technique was used for determination of the antibiotic susceptibility of bacteria. Eighteen antibiotics were tested including: Ciprofloxacin, Novobiocin, Streptomycin, Hygromycin, Chloramphenicol, Tetracycline, Trimethoprim, Carbenicillin, G418, Neomycin, Ampicillin, Vancomycin, Geneticin, Rifampicin, Spectinomycin, Apramycin, Ticarcillin and Kanamycin.

Antibiotics and chemical agents were diluted in sterile distilled water to prepare the solutions at the specified working concentrations. The antibiotics (Sigma Aldrich, Saint-Quentin-Fallavier, France) were used at the working concentrations of the following ranges: Ciprofloxacin (from 0.05 to 2.5 mg/mL), Novobiocin (from 0.748 to 37.4 mg/mL), Streptomycin (from 0.6 to 30 mg/mL), Hygromycin, Carbenicillin, G418, Neomycin, Ampicillin (from 2 to 100 mg/mL), Chloramphenicol (from 0.4 to 20 mg/mL), Tetracycline, Trimethoprim (from 0.5 to 25 mg/mL), Vancomycin (from 0.58 to 28.86 mg/mL), Geneticin, Kanamycin (from 0.8 to 40 mg/mL), Rifampicin (from 1.8 to 90 mg/mL), Spectinomycin (from 6 to 300 mg/mL), Apramycin (from 1 to 50 mg/mL) and Ticarcillin (250 mg/mL).

Chemical agents used in the experiment were: hydrogen peroxide (H_2_O_2_), magnesium carbonate (MgCO_3_), benzoic acid (C_7_H_6_O_2_), manganese sulphate monohydrate (MnSO_4_*H_2_O), sodium molybdate (Na_2_MoO_4_), zinc sulfate heptahydrate (ZnSO_4_*7H_2_O), iron (III) chloride (FeCl_3_), copper (II) sulfate pentahydrate (CuSO_4_*5H_2_O), ethylenediaminetetraacetic acid (EDTA; C_10_H_16_N_2_O_8_), iron (II) citrate (C_12_H_30_Fe_3_O_24_), potassium iodide (KI), potassium sulfate (K_2_SO_4_), p-hydroxybenzoic acid (C_7_H_6_O_3_), polyoxyethylenesorbitan monolaurate (tween; C_58_H_114_O_26_), mineral oil, and sodium dodecyl sulfate (SDS; C_12_H_25_NaO_4_S). Solutions of different chemical agents were prepared at the concentrations from 1 mg/ml to 50 mg/ml with the exception of hydrogen peroxide and dodecyl sulfate, which were used at the concentration from 0.6 to 30% and 0.004 to 20%, respectively. Antibiotic and chemical agents were added on the discs. The discs were placed on the King’s A media with growing bacterial cultures. To determine the efficiency of an individual antibiotic and chemical agent and their effective concentrations, the zone diameters were measured after overnight incubation at 37 °C. We measured the diameter of the growth inhibition zone [circular zone around an antibiotic disc with no visible bacterial growth (Figure S1)]. The diameter of the zone of inhibition of the test isolates were recorded. Some strains exhibited an inner area of light growth immediately around the antibiotic disc with an obvious area of inhibition outside this hazy growth. In these instances, the outer zone of inhibition was measured. The most obvious zone of inhibition was measured in those instances when several concentric zones were observed around an antibiotic disc.

## 3. Results

### 3.1. Susceptibility of Different Olive Cultivars to Olive Knot Disease

Different cultivars showed diverse intensities of symptoms for olive knot disease and were classified in six categories according to percentage of area affected (Table 1). We found no symptoms of disease in three Italian cultivars, Coratina, Favarol, and Leccino. The highest percentages of area covered by tumorous overgrowths ranging from 60–80% (category 5) and from 80–100% (category 6) were found for Frantoio, Lastovka, Santa Catarina and Chemlali. The Tunisian cultivar Chemlali was the most affected cultivar with the highest incidence of symptoms (category 6). Together with cultivars Fasolina, Grignan, Moraiolo and Sigoise, trees of Oblica, which is the most widespread cultivar in Croatia, showed very low percentage of area covered by symptoms ranging from 1-20% (category 2).

### 3.2. Genome Analysis of the Pseudomonas ST1 and Pantoea Paga 

Plants of Lastovka and Oblica, the most representative olive cultivars grown along the eastern coast of the Adriatic area were used to isolate and identify causal agent of gall formation that could coevolve with olive plants. Fresh galls from different plants where surface sterilized in ethanol, sliced by sterile scalpel and placed on King’s agar plates. We cultured two visually different types of colonies after two days of incubation at room temperature. Total DNA was isolated from twelve colonies and part of the 16sRNA gene was amplified and sequenced. Blast results of sequence analysis of the 16sRNA genes from twelve random isolated colonies show presence of two groups of bacteria belonging to *Pseudomonas* and *Pantoea* genera.

Genomes of *Pantoea* paga and *Pseudomonas* ST1 isolates were sequenced using ilumina NGS technology end each genome was assembled for genome analysis [37,38].

**Figure 1:**
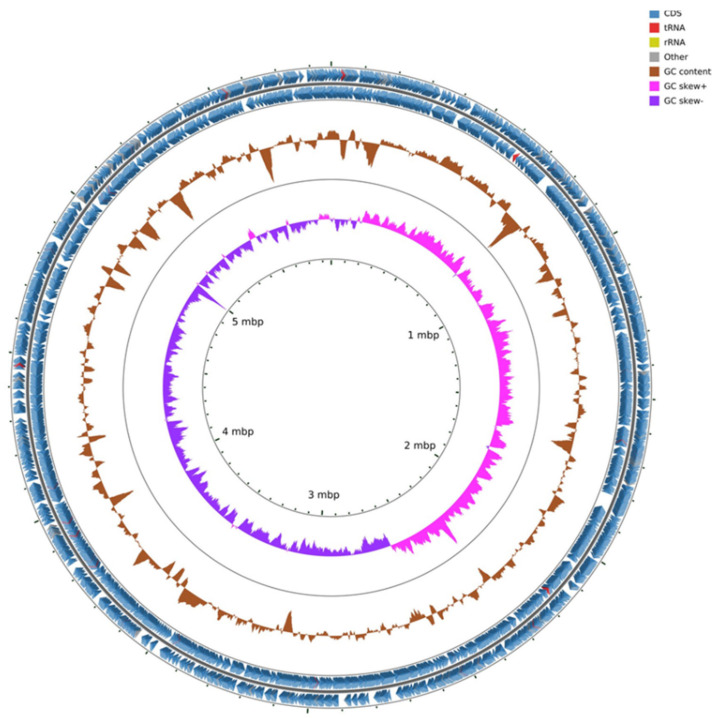
Genome map of *Pseudomonas* ST1 strain with coding sequence (CDS), transfer RNA (tRNA), ribosomal RNA (rRNA), GC content, and inner cycle scale.

**Figure 2:**
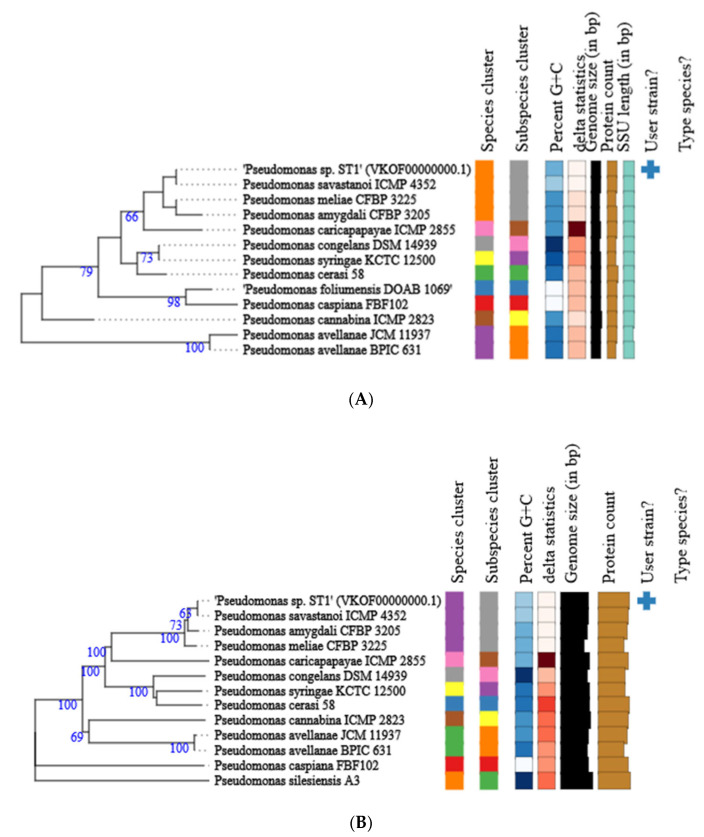
Phylogenetic tree of *Pseudomonas* ST1 strain calculated from 16S rDNA gene sequences (**A**); phylogenetic tree of *Pseudomonas* ST1 strain calculated from whole genome sequences (**B**). Different colors within each column indicate different minimum, maximum and intermediate values; and +represents user strain.

The genome of *Pseudomonas* ST1 has 6.1 Mbps, genome coding for 6271 genomic objects and 58% of GC contents (Figure 1). Phylogenetic analysis of the whole genome and 16S rRNA gene sequence of *Pseudomonas* ST1 show that this strain belongs to known species *Pseudomonas amygdali* (Figure 2A,B).

**Figure 3:**
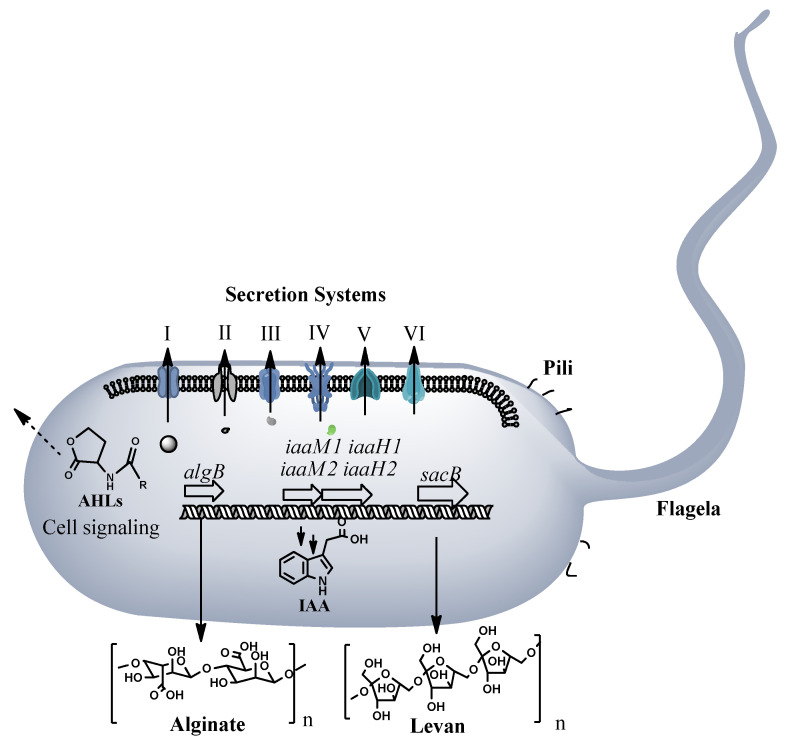
Pathogenicity and virulence factors of *Pseudomonas* ST1 strain, secretion system (I–VI), motility factors, pili and flagella, alginate and levan biosynthesis, IAA hormone biosynthesis and signaling molecule biosynthesis. For full list of conserved virulence related genes and proteins see Table S1.

*Pseudomonas* ST1 is a motile bacterium as confirmed under light microscopy observation and presence of flagellum and pili biosynthesis genes in the genome. Genome analysis shows presence of six secretion systems, from TSS1 to TSS6, and an alginate biosynthesis operon related to production of extracellular polysaccharides necessary to host attachment and virulence. The global activator, two component system *gacS* is also present in the genome and could regulate not only alginate production but also homo serine lactone production, toxin production and other virulence related genes and operons (Figure 3 and Table S1). Presence of two operons with genes (*iaaM1*, *iaaH1*, *iaaM2,* and *iaaH2*) involved in indole acetic acid (IAA) biosynthesis indicates potential for the strain to produce plant hormone. Eight genome clusters coding for secondary metabolites are present in the genome. Most of them are nonribosomal peptides synthetases (NRPS) involved in biosynthesis of secondary metabolites. Based on homology searches, these clusters can be involved in biosynthesis of antibiotics, signaling molecules, siderophores, toxins, and storage materials (Table S2).

Whole genome homology searches show similarity of *Pseudomonas* ST1 isolates to the *Pseudomonas savastanoi* ICMP. Two strains share 5442 common genes and each strain has strain-specific genes. *Pseudomonas* ST1 has 568 strain-specific genes in comparison and *Pseudomonas savastanoi* that has 434 specific genes (Table S3 and Figure S2).

**Figure 4:**
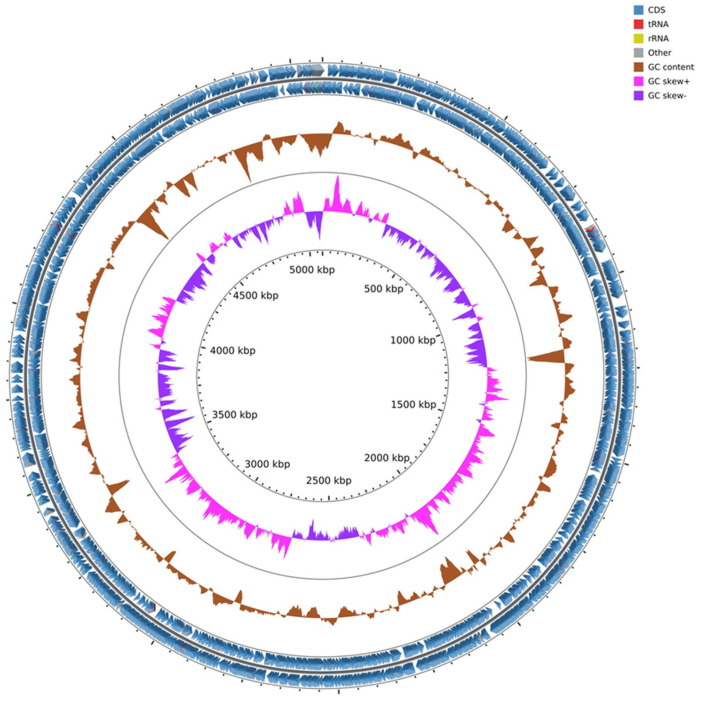
Genome map of *Pantoea* paga strain with coding sequence (CDS), transfer RNA (tRNA), ribosomal RNA (rRNA), GC content, and inner cycle scale.

The *Pantoea* paga genome is 5.08 Mbps with 54.58% of G + C contents. The genome has 4777 coding sequences, one ribosomal operon coding for 16s, 23s, and 5s rRNA genes, 70 tRNA genes and 91 misc_RNA (Figure 4).

The genome has T1SS, T3SS, and T5SS genes for secretion systems, *focA* gene for pilin biosynthesis, *hlyF* gene for hemolysin, and only one *hopD1* gene related to pathogenicity. Seven genome regions contain genes for secondary metabolite biosynthesis such as carotenoids, zeaxantinins, siderophores, and homoserine lactone. The *Pantoea* paga strain has *mcb* bacteriocin biosynthesis operon containing genes involved in biosynthesis, maturation, modification, and secretion of antimicrobial peptide.

**Figure 5:**
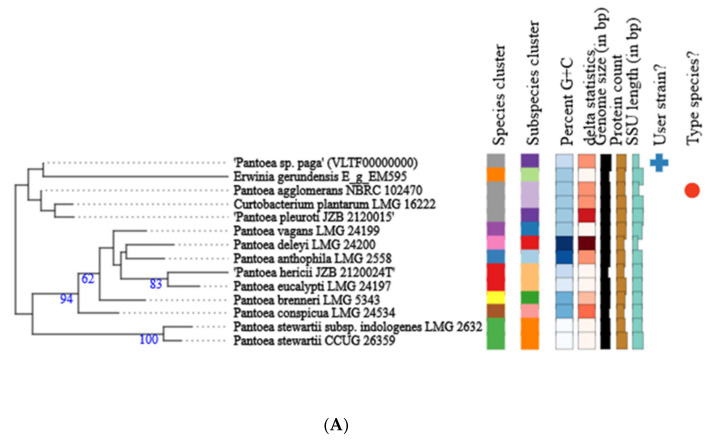

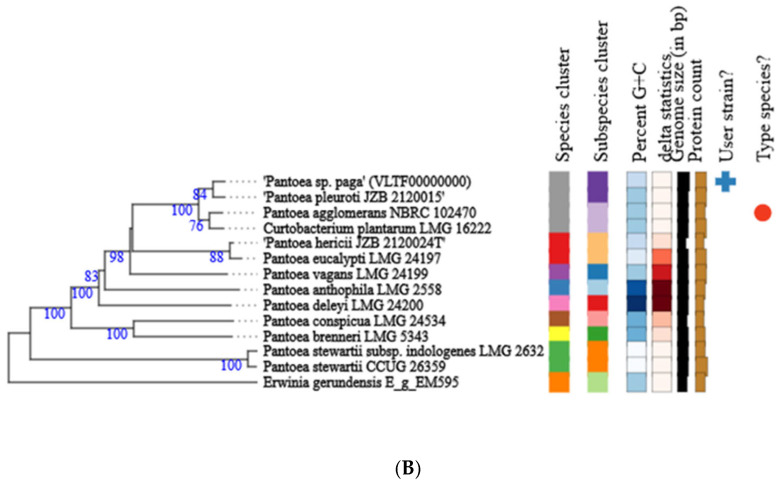
Phylogenetic tree of *Pantoea* paga strain calculated from 16S rDNA gene sequences (**A**); phylogenetic tree of *Pantoea* paga strain calculated from whole genome sequences (**B**). Different colors within each column indicate different minimum, maximum and intermediate values; + represents user strain and red dot indicates type species.

Phylogenetic analysis of the whole genome and 16S rRNA gene sequence of *Pantoea* paga shows that this strain belongs to the known species *Pantoea agglomerans* (Figure 5A,B).

**Figure 6:**
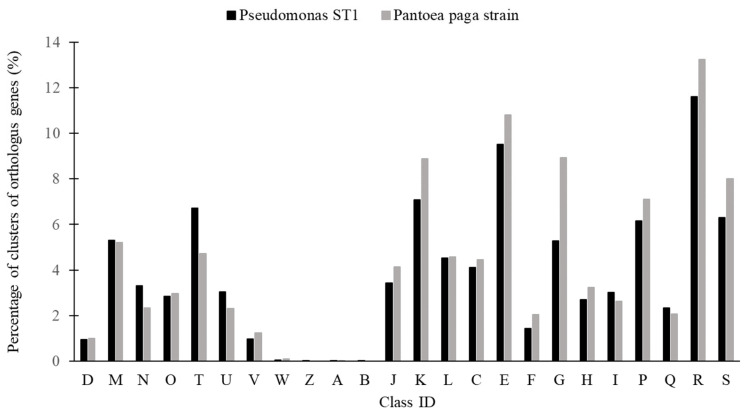
Percentage of clusters of orthologus genes (%) in *Pseudomonas* ST1 strain and *Pantoea* paga strain. Letters on the x-axis correspond to different groups of functional genes: D—cell cycle control, cell division, chromosome partitioning; M—cell wall/membrane/envelope biogenesis; N—cell motility; O—posttranslational modification, protein turnover, chaperones; T—signal transduction mechanisms; U—intracellular trafficking, secretion, and vesicular transport; V—defense mechanisms; W—extracellular structures; Z—cytoskeleton; A—RNA processing and modification; B—chromatin structure and dynamics; J—translation, ribosomal structure, and biogenesis; K—transcription; L—replication, recombination, and repair; C—energy production and conversion; E—amino acid transport and metabolism; F—nucleotide transport and metabolism; G—carbohydrate transport and metabolism; H—coenzyme transport and metabolism; I—lipid transport and metabolism; P—inorganic ion transport and metabolism; Q—secondary metabolites biosynthesis, transport and catabolism; R—general function prediction only; and S—function unknown.

The statistic distribution of the protein coding genes of the two genomes within the clusters of orthologous groups of functional categories is shown in Figure 6 [40].

### 3.3. Pathogenicity of Pseudomonas ST1 and Pantoea Paga

**Figure 7:**
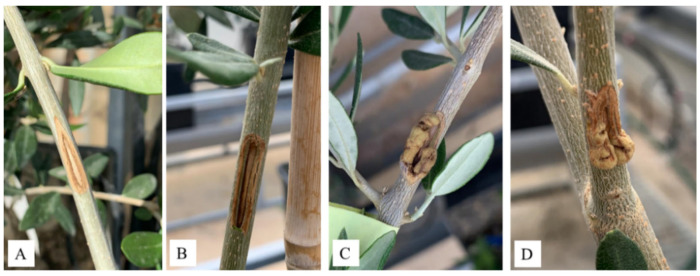
Appearance of symptoms of olive knot disease six months post inoculation by *Pseudomonas* ST1 and *Pantoea* paga strains (**A**) control plants; (**B**) plants inoculated with *Pantoea* paga strain; (**C**) plants inoculated with *Pseudomonas* ST1 strain; and (**D**) plants inoculated with a mixture of both bacteria.

To verify the causative agent of knot formation we inoculated healthy olive plants with isolated bacterial species alone and in a mixture of the two bacterial isolates. Pure culture of two strains were mixed and inoculated on healthy olive plants. Six months post inoculation of bacteria we observed formation of galls on plants tissues and compared symptoms with control plants (Figure 7A–D). We observed formation of galls when *Pseudomonas* ST1 strain was used as inoculant either alone or in a mixture with *Pantoea* sp. paga strain (Figure 7C,D). There was no gall formation on the plants where *Pantoea* paga strain was applied alone (Figure 7B).

**Figure 8:**
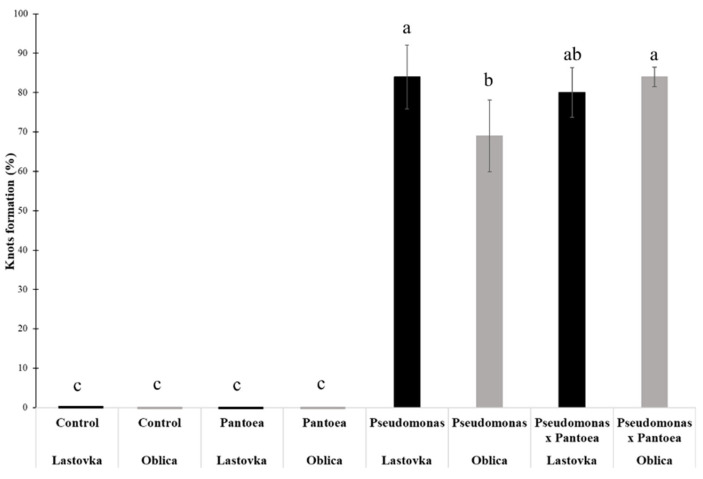
Percentage of knots formation on Oblica and Lastovka cultivars inoculated with *Pantoea* paga or *Pseudomonas* ST1 strains used alone or with mixture of two strains compared to non-inoculated control plants. The results are expressed as the means ± SE. Different lowercase letters indicate significant differences according to the least significant difference test at *p* < 0.05.

Total DNA was isolated from infected areas of plant tissues. Presence of two bacteria was confirmed by PCR amplification of 16s rDNA genes and PCR amplification of strain specific genes using specific pairs of primers followed by amplicon sequencing. Formation of knots and presence of *Pseudomonas* ST1 strain was confirmed in all tested knots. We conclude that the isolate of *Pseudomonas* sp. alone is the causative agent of knot formation on olive plants (Figure 8). *Pantoea* sp. cells inoculated alone on olive plants did not induce formation of galls. Lastovka showed higher level of knot formation compared to Oblica when inoculated with *Pseudomonas* ST1 strain (Figure 8). A mixture of two bacteria enhanced the development of knots as observed in Oblica.

### 3.4. Susceptibility of Two Bacteria to Various Chemical Agents and Antibiotics

The antibacterial activities of the antibiotics and chemical agents in different concentrations against the *Pseudomonas* ST1 and *Pantoea* paga strains were examined for the presence or absence of the inhibition zones by the agar disc diffusion method. Sensitivity values of each antibiotic and chemical agent used for antibacterial activity are shown in Table 2.

*Pseudomonas* ST1 strain showed the highest susceptibility to all of the used antibiotics, while *Pantoea* paga strain showed reduced sensitivity to hygromycin and vancomycin. Both strains were resistant to iron (II) citrate, magnesium carbonate, mineral oil, p-hydroxybenzoic acid, polyoxyethylenesorbitan monolaurate, potassium iodide, and potassium sulfate. The sodium molybdate was not effective against *Pantoea* paga. The high efficiency of sodium dodecyl sulfate on *Pseudomonas* ST1 was confirmed. Both bacterial strains were very susceptible to copper salts. Hydrogen peroxide showed the highest activity against both bacteria and it is one of the best candidates in the protection of olive plants from the bacteria causing olive knot.

## 4. Discussion

*Pseudomonas savastanoi* and *Pantoea aglomerans* are recognized as causal agents of plant knot disease, causing aerial tumorous growth that evolve to necrosis [1,3,4,34,35,50]. Each bacterium alone or together can induce tumor formation on plant tissue [10,51].

The occurrence and intensity of symptoms in olive cultivars was assessed in the Olive Germplasm Bank of Croatia. In earlier studies, the two most common bacterial species associated with the olive-knot microbiome were isolated from the knots of olive trees grown in Croatia and their genomes have been sequenced [37,38]. Two bacterial strains of *Pseudomonas* [52] and *Pantoea* [53] are known as causative agents of knot formation on plants. In silico analysis of *Pseudomonas* ST1 genome shows presence of all virulence genes necessary for infection such as type T3SS secretion system, protected polymers production, alginate, levan, and production of the phytohormone IAA [54,55]. The strain has two putative operons for production of IAA that could be related to high infectivity of this endemic strain in knot formation. This strain shares a high proportion of genes in common with other *Pseudomonas* strains. However, strain ST1 has 568 specific genes with unknown function. These genes may contribute to its specific ecological niche. It possesses three gene clusters coding non-ribosomal peptide synthetase that could synthetize bicornutin, rhizomide, and mangotoxin. According to phylogenetic analyses the *Pseudomonas* ST1 strain belongs to the *Pseudomonas amygdali* species.

In contrast, *Pantoea* paga has no panel of virulence genes in its genome. This strain belongs to the *Pantoea agglomerans* species and probably uses *Pseudomonas* ST1 to invade plant tissue. It contains antimicrobial genes involved in microcin biosynthesis that is known as antibacterial peptide inhibiting DNA gyrase [56]. Therefore, the presence of *Pantoea* paga can affect the growth of other bacteria.

The genomes were further analyzed in the present study to determine differences between the two bacteria. These two bacteria were used in the new experiment to determine whether their co-infection intensifies the symptoms of olive knot disease in two olive cultivars Lastovka and Oblica and to determine whether each of them is sufficient to cause the symptoms. *Pseudomonas* ST1 is the causative agent of knot formation as it was shown in the experiment where the healthy olive plants were inoculated with two bacteria. Inoculation with the *Pantoea* strain did not induce knot formation when inoculated alone; however, join inoculation with *Pseudomonas* ST1 strain increased the percentage of knot formation in Oblica cultivar. The differences in occurrence and intensity of symptoms in cultivars Oblica and Lastovka were less pronounced in the greenhouse experiment, where the young plants were artificially infected compared to the evaluation carried out in the collection orchard, where the knots were formed over the longer period before the assessment and without our interference.

To avoid the spread of *P. savastanoi* or other pathogens, it is important to develop strain specific identification. Here we successfully used strain specific PCR oligonucleotide primers for the detection of two bacteria. Furthermore, in order to improve measures to control the spread of this disease in the process of propagation of olive plant material, the efficiency of antibiotics and other chemical agents in control of pathogens has been investigated. Our results provide new insights on the epidemiology of this disease and enable *Pseudomonas* and *Pantoea* control.

## Figures and Tables

**Table 1:** The classification of 21 olive cultivars in six categories according to their tolerance to olive knot disease (from category 1–the highest tolerance to category 6–the lowest tolerance). The categories correspond to percentages of area affected by disease (1. Nil: 0; 2. Very low: 1–20%; 3. Low: 20–40%; 4. Medium: 40–60%; 5. High: 60–80%; 6. Very high: 80–100% of area affected).

Category					
1	2	3	4	5	6
Cultivar					
Coratina	Fasolina	Canino	Drobnica	Frantoio	Chemlali
Favarol	Grignan	Koroneiki	Pendolino	Lastovka	
Leccino	Moraiolo	Levantinka	P. marocaine	S. Catarina	
	Oblica	Maurino	Taggiasca		
	Sigoise	Rosciola			

Low incidence of symptoms ranging from 20–40% of area affected (category 3) was found for Canino, Koroneiki, Levantinka, Maurino, and Rosciola while medium percentages (40–60%) were found for Drobnica, Pendolino, Picholine marocaine, and Taggiasca.

**Table 2:** In vitro antibacterial activity of the antibiotics and chemical agents tested against *Pseudomonas* ST1 and *Pantoea* paga strains. Tested concentrations are shown in mg/mL or percentage (%).

Antibiotic	Pseudomonas ST1 Sensitivity	Pantoea PAGA Sensitivity
Ampicillin	2	2
Apramycin	1	1
Carbenicillin	2	2
Chloramphenicol	0.4	0.4
Ciprofloxacin	0.05	0.05
G418	2	2
Geneticin	0.8	0.8
Hygromycin	2	10
Kanamycin	0.8	0.8
Neomycin	2	2
Novobiocin	0.748	0.748
Rifampicin	1.8	1.8
Spectinomycin	6	6
Streptomycin	0.6	0.6
Tetracycline	0.5	0.5
Ticarcillin	250	250
Trimethoprim	0.5	0.5
Vancomycin	0.58	28.86
**Chemical Agent**		
Benzoic acid (C_7_H_6_O_2_)	50	10
Copper (II) acetate	5	5
Copper (II) sulfate pentahydrate (CuSO_4_*5H_2_O)	5	5
Ethylenediaminetetraacetic acid (EDTA; C_10_H_16_N_2_O_8_)	25	10
Hydrogen peroxide (H_2_O_2_)	0.6%	0.6%
Iron (II) citrate (C_12_H_30_Fe_3_O_24_)	Resistant	Resistant
Iron (III) chloride (FeCl_3_)	1	1
Magnesium carbonate (MgCO_3_)	Resistant	Resistant
Manganese sulphate monohydrate (MnSO_4_*H_2_O)	10	50
Mineral oil	Resistant	Resistant
p-hydroxybenzoic acid (C_7_H_6_O_3_)	Resistant	Resistant
Polyoxyethylenesorbitan monolaurate (tween; C_58_H_114_O_26_)	Resistant	Resistant
Potassium iodide (KI)	Resistant	Resistant
Potassium sulfate (K_2_SO_4_)	Resistant	Resistant
Sodium dodecyl sulfate(SDS; C_12_H_25_NaO_4_S)	0.004%	20%
Sodium molybdate (Na_2_MoO_4_)	50	Resistant
Zinc sulfate heptahydrate(ZnSO_4_*7 H_2_O)	1	1

## Data Availability

The original contributions generated for this study are included in the article/Supplementary Material; further inquiries can be directed to the corresponding author.

## Supplementary Materials

The following supporting information can be downloaded at: https://www.mdpi.com/article/10.3390/microorganisms10081529/s1. Figure S1: Disc diffusion method for antibiotic and chemical agent’s susceptibility testing. Figure S2: Venn diagram showing two genomes (*Pseudomonas* ST1 and *Pseudomonas savastanoi* pv. *savastanoi* PVFi1) with core genome and strain specific genes (Table S3). Table S1: Conserved *Pseudomonas* ST1 virulence genes. Table S2: Cluster of genes for biosynthesis of secondary metabolites; Table S3: Strain specific and common genes in *Pseudomonas* ST1.
