# Supplementary material for: Pseudomonas ST1 and Pantoea Paga Strains Cohabit in Olive Knots
Source: Microorganisms. 2022 Jul 28;10(8):1529. doi: 10.3390/microorganisms10081529 (PMC9414602; doi:10.3390/microorganisms10081529)
Supplement: Supplementary file 1 [file microorganisms-10-01529-s001.zip › Supplement Figure S1.pptx]

## Slide 1
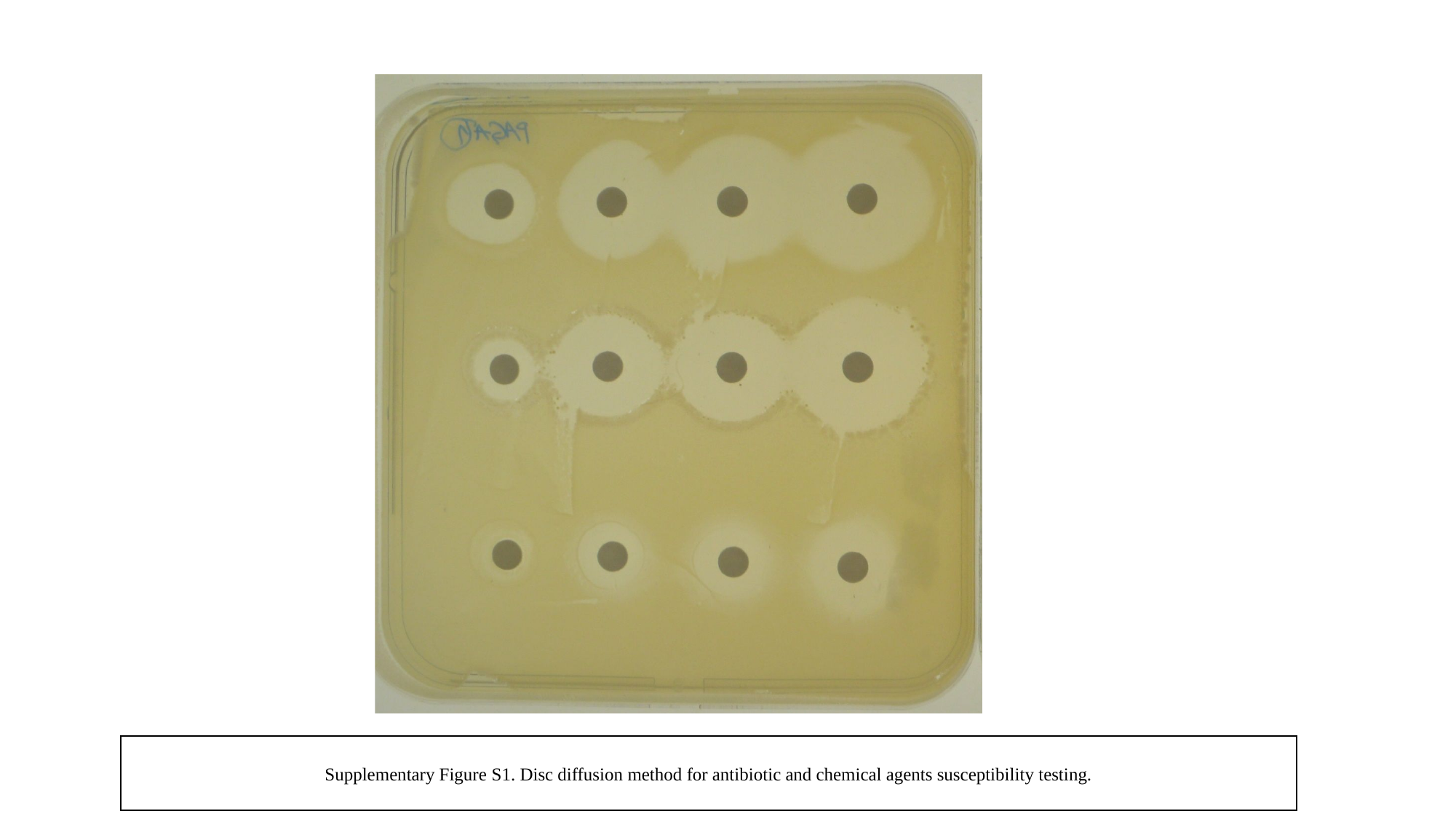

Supplementary Figure S1. Disc diffusion method for antibiotic and chemical agents susceptibility testing.
