# Supplementary material for: Pseudomonas ST1 and Pantoea Paga Strains Cohabit in Olive Knots
Source: Microorganisms. 2022 Jul 28;10(8):1529. doi: 10.3390/microorganisms10081529 (PMC9414602; doi:10.3390/microorganisms10081529)
Supplement: Supplementary file 1 [file microorganisms-10-01529-s001.zip › Supplement Figure S2.pdf]

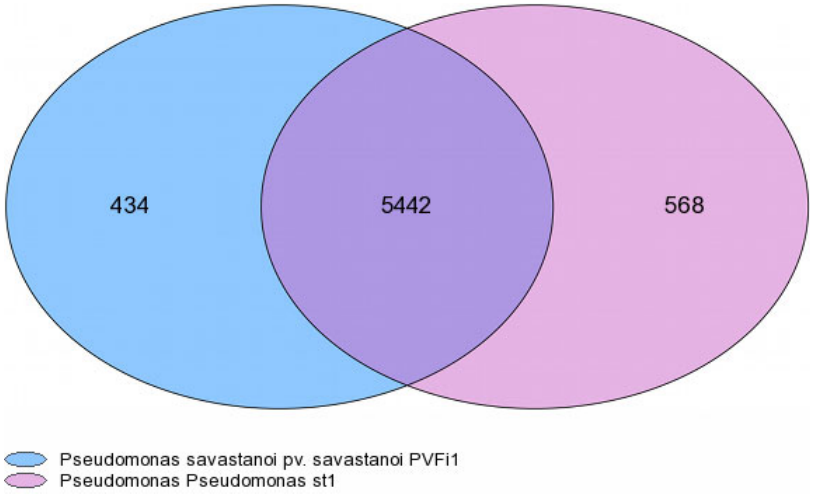

Figure S2. Venn diagram showing two genomes (*Pseudomonas* ST1 and *Pseudomonas savastanoi* pv. *savastanoi* PVFi1) with core genome and strain specific genes (Table S3).
